# Editorial: Advances in phenomics, metabolomics, and genomics for precision breeding of ornamental horticultural crops

**DOI:** 10.3389/fpls.2026.1936025

**Published:** 2026-09-07

**Authors:** Mihaiela Cornea-Cipcigan, Jameel M. Al-Khayri, Alexandru Nicolescu, Muneeb Ahmad Wani

**Affiliations:** 1 Department of Clinical and Paraclinical Sciences, Faculty of Veterinary Medicine, University of Agricultural Sciences and Veterinary Medicine Cluj-Napoca, Cluj-Napoca, Romania; 2 Department of Agricultural Biotechnology, College of Agriculture and Food Sciences, King Faisal University, Al-Ahsa, Saudi Arabia; 3 National Institute for Research and Development of Isotopic and Molecular Technologies, Cluj-Napoca, Romania; 4 Division of Floriculture and Landscape Architecture, Sher-e-Kashmir University of Agricultural Sciences and Technology of Kashmir (SKUAST-K), Srinagar, Jammu and Kashmir, India

**Keywords:** metabolomics floral traits, molecular markers, ornamental plant genomics, phenomics technologies, precision breeding

## Introduction

Precision breeding has fundamentally transformed ornamental crop improvement by shifting selection from morphology-based approaches to data-driven, multi-omics frameworks. The convergence of phenomics, metabolomics, and genomics now enables a systems-level understanding of trait architecture, which is critical for improving ornamental value as well as resilience under environmental constraints. In ornamental species, target traits extend beyond aesthetics to include floral color, volatile fragrance profiles, pathogen resistance, and, increasingly, tolerance to abiotic stresses such as drought, salinity, heat, and low irradiance. Abiotic stress tolerance has become a priority as climate variability intensifies and water resources for horticulture become limited. Recent advances in plant biotechnology, molecular genetics, and rhizosphere ecology are reshaping our understanding of crop improvement strategies across both food and ornamental species. Metabolomics offers a functional lens on the biochemical basis of ornamental traits, particularly floral scent, pigmentation, and secondary metabolites that define market value. Profiling via mass spectrometry MS and nuclear magnetic resonance NMR resolves complex biosynthetic pathways for volatile organic compounds VOCs and anthocyanins. In ornamentals such as rose, chrysanthemum, cyclamen, gerbera, and petunia, metabolomic mapping of anthocyanin networks has guided breeding for improved color stability and intensity. Equally important, metabolomics is a key tool for abiotic stress physiology. Stress-induced shifts in phenolic acids, flavonoids, polyamines, sugars, and antioxidant metabolites can serve as biomarkers for drought or salinity tolerance. For example, drought-tolerant rose genotypes exhibit coordinated accumulation of phenolic compounds, glutathione metabolism intermediates, and jasmonate signaling metabolites, whereas other species rely on proline or anthocyanin accumulation. Identifying such stress-associated metabolites enables breeders to select resilient cultivars and link metabolic QTLs to genomic loci for marker-assisted improvement. Plant genotyping has evolved from PCR-based markers such as SSRs, SRAP, SCoT, RAPD, to high-resolution, genome-wide approaches. Next-generation sequencing NGS technologies, including RAD-seq, GBS, and whole-genome re-sequencing, now support simultaneous SNP discovery and genotyping, facilitating high-density linkage mapping, QTL detection, and genomic selection in ornamentals. Microarray and SNP-panel technologies further enable thousands of markers to be assayed in parallel. Integrating these modern tools with phenomic and metabolomic datasets enhances genetic mapping, marker-assisted breeding MAS, and trait association, thereby shortening breeding cycles and improving conservation of genetic resources.

**Figure 1:**
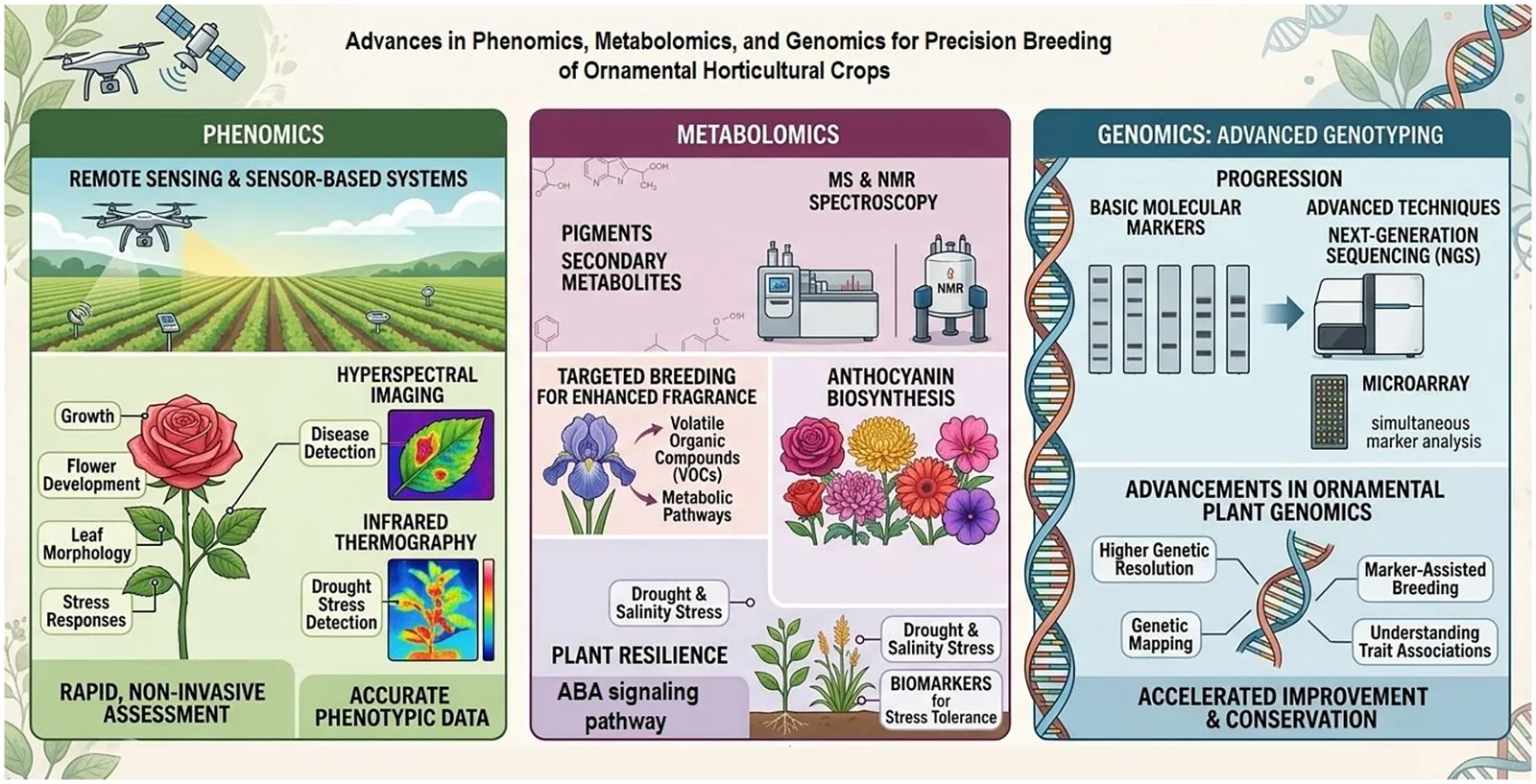
Illustrative overview of the topic - Advances in phenomics, metabolomics, and genomics for precision breeding of ornamental horticultural crops.

To highlight the broad applicability of such modeling approaches, we have compiled a collection of research and review articles on the topic of precision breeding that address advances in phenomics, metabolomics, and genomics that address key questions in food and ornamental crops (**Figure 1**). A total of 6 articles by 36 authors cover a broad spectrum of the topic. The studies reviewed here collectively highlight how emerging technologies, including somatic embryogenesis, CRISPR/Cas9 gene editing, molecular marker-assisted diversity analysis, genomic resource development, and multi-omics approaches are transforming the breeding and management of economically important food and ornamental crops such as peony (*Paeonia* sp.), potato (*Solanum tuberosum*), bearded iris (*Iris germanica*), and ornamental dogwoods (*Cornus* sp.). Together, these investigations demonstrate that future breeding success will depend on integrating genetic innovation with ecological understanding and sustainable cultivation practices.

## Somatic embryogenesis and genetic transformation: unlocking the potential of peony improvement

*Paeonia* sp. (peony) represents one of the world’s most valuable ornamental crops, prized for its aesthetic appeal, extended vase life, and expanding commercial significance in horticulture and nutraceutical markets. However, conventional propagation by division remains slow, labor-intensive, and constrained by seasonal cycles, limiting the rapid dissemination of elite cultivars and the implementation of precision breeding strategies. Somatic embryogenesis SE has emerged as a promising biotechnological solution, offering opportunities for large-scale clonal propagation, cryopreservation, and genetic transformation with high genetic fidelity.Recent advances in peony biotechnology have clarified several factors influencing SE efficiency (Elhiti et al., 2013). Among these, genotype remains the most decisive determinant of successful embryogenic induction. Significant variation exists among cultivars, with many commercially important genotypes exhibiting strong recalcitrance to tissue culture. Explant selection also plays a critical role, as developmental stage and tissue origin directly influence embryogenic competence (Jia et al., 2022). Parallel advances in genetic transformation technologies have expanded opportunities for functional genomics in peony. Stable transformation systems utilizing Agrobacterium-mediated approaches have demonstrated feasibility, while transient expression techniques, including Agrobacterium infiltration and virus-induced gene silencing (VIGS), have accelerated gene function studies. Nevertheless, transformation efficiency remains low, regeneration systems are often incomplete, and genotype dependency continues to restrict applicability. The article by Fu et al., evaluated the current state of SE and genetic transformation in Paeonia, a traditional ornamental flower and economically important crop that requires efficient propagation and targeted breeding for industrial scalability. They have assessed the key determinants of SE efficiency, including the decisive role of genotype, explant developmental stage and origin, plant growth regulator combinations, and controlled culture conditions, and identified genotype-dependent recalcitrance as the primary constraint. The authors have also analyzed critical bottlenecks limiting commercial SE application, with particular emphasis on browning caused by phenolic oxidation and hyperhydricity, and discussed physiological and molecular factors underlying these disorders. In addition, they have reviewed advances in *Paeonia* transformation systems, evaluating both stable *Agrobacterium*-mediated methods and transient approaches such as *Agrobacterium* infiltration and virus-induced gene silencing VIGS for functional genomics. Based on this evaluation, the authors conclude that despite progress, low regeneration efficiency, incomplete transformation pipelines, and broad genotype dependence persist, and they propose that integrating optimized tissue culture systems with emerging gene editing technologies will be essential to overcome these barriers and develop new *Paeonia* cultivars with improved industrial and market potential. Future progress will likely depend on combining optimized tissue culture systems with next-generation gene editing technologies. Such integration could enable precise trait modification while overcoming limitations associated with traditional breeding, ultimately supporting the development of novel peony cultivars with improved ornamental value, disease resistance, and production efficiency.

## Challenges in salt tolerance screening of ornamental peony germplasm

Soil salinization is a growing global constraint on agriculture, with over 900 million hectares of land affected worldwide. It acts as a major abiotic stress that limits plant growth by triggering three interrelated types of damage: osmotic stress, ionic stress, and oxidative stress (Dehnavi et al., 2023). Ornamental flowering plants have received comparatively little attention, despite their economic and cultural value. Herbaceous peony *Paeonia lactiflora* Pall. is one such case. With a cultivation history of more than 4,000 years in China, it is valued for large, colorful flowers, strong adaptability, and high landscaping and cut-flower market value. It also has medicinal importance because its main bioactive compound, paeoniflorin, shows antioxidant, anti-inflammatory, and anticancer activities. However, soil salinity strongly limits large-scale cultivation and landscape use of peony because salt stress reduces growth, development, and ornamental quality (Jiang et al., 2018). Understanding how peony responds physiologically to salt and evaluating available germplasm are thus critical for expanding its cultivation into saline-affected areas. The study by Lu et al., has addressed the impact of soil salinization on herbaceous peony (*P. lactiflora*), a widely cultivated ornamental flower in China, by screening for relative salt tolerance among 20 cultivars. They have evaluated salt response using a severe stress regime of 400 mM NaCl applied five times over 15 days, with a non-saline control for phenotypic reference, and measured seven pre-treatment morphological indices alongside post-treatment phenotypic and physiological traits (Weng et al., 2021). Based on phenotypic scoring and cluster analysis, the authors have identified ‘Red Charm’, ‘Bartzella’, and ‘Sarah Bernhardt’ as the most salt-tolerant cultivars under severe stress. They have further determined, through correlation and stepwise regression analyses, that leaf thickness and stem diameter are potential preliminary morphological indicators for rapid salt tolerance assessment, with higher values associated with greater tolerance. Physiologically, the authors have shown that tolerant cultivars maintained higher antioxidant enzyme activities and exhibited significantly lower relative electrolyte leakage and MDA content than sensitive cultivars, while Fv/Fm declined markedly with decreasing salt tolerance. Overall, the authors provide exploratory evidence that specific morphological and photosynthetic parameters can support rapid screening of salt-tolerant herbaceous peony germplasm.

## Propagation and molecular mechanisms of dogwood and CRISPR-based technology for potato tuber formation

While ornamental crops highlight the importance of aesthetic traits, food crops underscore the necessity of improving productivity and food security. Potato remains one of the world’s most important staple crops, contributing significantly to global nutrition and economic development. Understanding the molecular mechanisms regulating tuber formation is therefore of considerable agricultural importance. Hamm et al. reviewed the big-bracted *Benthamidia* dogwood clade within *Cornus* of family Cornaceae, Cornales, a lineage of small- to medium-sized deciduous trees valued for their showy spring floral bract display and phylogenetic importance as one of the earliest diverging asterid groups. They have evaluated three economically significant North American ornamental species, such as flowering dogwood *C. florida*, kousa dogwood *C. kousa*, and Pacific dogwood *C. nuttallii*, along with the more than 130 cultivars developed from them, and identified that persistent research gaps continue to limit both fundamental investigations and commercial breeding progress. The authors have synthesized current knowledge on the phylogenetic relationships and biogeographic history that shape species diversification, the key aspects of plant biology together with the major insect pests and pathogens that constrain landscape performance and commercialization, and the historical propagation methods that have underpinned nursery production. They have also examined the status of genetic and genomic resources, including marker systems, transcriptomic datasets, and reference genomes, and how these tools have been applied to characterize diversity, trace pedigrees, and inform selection. Across these themes, the authors have highlighted critical bottlenecks, articulated priority research gaps, and proposed future directions needed to advance basic research and enable more efficient breeding of big-bracted ornamental dogwoods.

CRISPR/Cas9 technology has emerged as a transformative genome editing tool in crop improvement due to its simplicity, precision, and cost-effectiveness. It has been successfully deployed across diverse plant species to modify traits related to yield, quality, and stress resistance, and it is especially valuable for clonally propagated, highly heterozygous, and polyploid crops such as potato, where conventional breeding is slow and genetically constrained. By enabling targeted modification without lengthy backcrossing, CRISPR/Cas9 offers a direct route to accelerate potato breeding and address key agronomic challenges.

Liu et al. investigated the functional role of StSN2, a gibberellic acid-stimulated Arabidopsis GASA family antimicrobial peptide, in potato tuber formation, addressing a key gap in the molecular regulation of yield. Using CRISPR/Cas9-mediated gene editing, they have evaluated how loss of StSN2 expression affects tuber development and demonstrated that StSN2 deletion delays stolon formation by approximately 14 days and reduces overall yield by 20-30%. The authors have combined bioinformatic and physiological approaches to characterize the StSN2 promoter, identified multiple hormone-responsive cis-elements, and confirmed through exogenous ABA and GA treatments that StSN2 is strongly induced by ABA. They have further elucidated the downstream mechanism by showing that StSN2 promotes tuberization via enhancement of ABA signaling, specifically by upregulating core pathway components StPYL1, StSnRK2.2/2.3/2.6, and StABI5, as supported by analyses of gene expression and enzyme activity during tuber development. By integrating genetic, molecular, and physiological data, the authors have established StSN2 as a critical positive regulator of potato tuber formation and provide a molecular basis for yield improvement through targeted manipulation of hormone signaling pathways (Bortesi and Fischer, 2015).

## Genetic diversity assessment as a foundation for breeding programs

Effective breeding programs depend on two complementary pillars: the technical capacity for genetic manipulation and a comprehensive, quantitative understanding of extant genetic diversity. Without detailed knowledge of allele distribution, population structure, and relatedness among accessions, selection efficiency declines and the risk of genetic erosion increases. The cultivated bearded iris *Iris germanica* L. provides a compelling case study of how molecular marker technologies reveal cryptic genetic relationships that morphology alone cannot resolve (Weber et al., 2020). Using both inter-simple sequence repeat ISSR and sequence-related amplified polymorphism SRAP markers, Bo et al. characterized 72 ISSR bands with 89.86% polymorphism and 693 SRAP bands with 96.54% polymorphism across cultivars. This high polymorphism indicates a substantial genetic reservoir available for hybridization and selection. Critically, SRAP markers exhibited superior discriminatory power compared to ISSR. SRAP clustering aligned more closely with phenotypic traits and geographic origins, enabling accurate grouping of accessions from the same region and with similar floral architecture. The combined ISSR and SRAP analysis effectively distinguished dwarf vs. tall growth habits and resolved closely related cultivar pairs such as ‘Antique Red’/’Indian Leader’ and ‘Bloodstone’/’Cherry Garden’. Such resolution is essential for germplasm management, core collection construction, and parent selection, as it allows breeders to avoid redundant crosses between genetically similar lines and to maximize heterosis by crossing divergent groups. These findings exemplify a broader principle for ornamental and agricultural species: characterizing genetic diversity is foundational to sustainable breeding (Makarevitch et al., 2003). Molecular markers serve dual functions: they preserve biodiversity by documenting unique genotypes for *ex situ* conservation, and they facilitate targeted improvement by guiding efficient crossing schemes. In vegetative propagated crops like *I. germanica*, where pedigree records are often incomplete, marker-based relatedness estimates reduce uncertainty and improve genetic gain per breeding cycle.

Collectively, these studies illustrate a transformative period in plant science. Advances in biotechnology, genomics, and systems biology are converging to create unprecedented opportunities for crop improvement Looking ahead, the future of crop improvement lies not in isolated technological advances but in their integration. Combining gene editing, genomic selection, advanced propagation systems, and ecological management strategies will enable the development of high-performing, resilient, and sustainable crop varieties. Such innovations are essential not only for enhancing ornamental plant industries but also for strengthening global food security and agricultural sustainability in an increasingly complex and resource-constrained world.

## References

[B1] Bortesi L. Fischer R. (2015). The CRISPR/Cas9 system for plant genome editing and beyond. Biotechnol. Adv. 33, 41–52. doi: 10.1016/j.bioteChadv.2014.12.006 25536441

[B2] Dehnavi A. R. Zahedi M. Piernik A. (2023). Understanding salinity stress responses in sorghum: exploring genotype variability and salt tolerance mechanisms. Front. Plant Sci. 14, 1296286. doi: 10.3389/fpls.2023.1296286 38269142 PMC10806974

[B3] Elhiti M. Stasolla C. Wang A. (2013). Molecular regulation of plant somatic embryogenesis. In Vitro Cell. Dev. Biology-Plant 49, 631–642. doi: 10.1007/s11627-013-9547-330311153

[B4] Jia M. Sun X. Chen M. Liu S. Zhou J. Peng X. (2022). Deciphering the microbial diversity associated with healthy and wilted Paeonia suffruticosa rhizosphere soil. Front. Microbiol. 13, 967601. doi: 10.3389/fmicb.2022.967601 36060757 PMC9432862

[B5] Jiang C. Ye K. Gao Y. Song Y. Mo J. (2018). Effects of salt stress on physiological indexes in 13 varieties of herbaceous peony. J. Northwest. Forestry. Univ. 33, 70–74. doi: 10.3969/j.issn.1001-7461.2018.02.11

[B6] Makarevitch I. Golovnina K. Scherbik S. Blinov A. (2003). Phylogenetic relationships of the siberian iris species inferred from noncoding chloroplast DNA sequences. Int. J. Plant Sci. 164, 229–236. doi: 10.1086/34616021668328

[B7] Weber T. Jakše J. Sladonja B. Hruševar D. Landeka N. Brana S. . (2020). Molecular study of selected taxonomically critical taxa of the genus Iris L. from the broader Alpine-Dinaric area. Plants 9, 1229. doi: 10.3390/plants9091229 32961899 PMC7570032

[B8] Weng J. Li P. Rehman A. Wang L. Gao X. Niu Q. (2021). Physiological response and evaluation of melon (*Cucumis melo* L.) germplasm resources under high temperature and humidity stress at seedling stage. Sci. Hortic. 288, 110317. doi: 10.1016/j.scienta.2021.11031742574925

